# Comparison of conventional reconstruction plate versus direct metal laser sintering plate: an in vitro mechanical characteristics study

**DOI:** 10.1186/s13018-017-0628-6

**Published:** 2017-09-02

**Authors:** Pusheng Xie, Hanbin Ouyang, Yuping Deng, Yang Yang, Jing Xu, Wenhua Huang

**Affiliations:** Guangdong Provincial Key laboratory of Medical Biomechanics, Department of Anatomy, School of Basic Medicine Science, Southern Medical University, 1023 ShaTai Rd, Guangzhou, 510515 People’s Republic of China

**Keywords:** Mechanical property, DMLS, Ti-6Al-4V, CNC, Reconstruction plate

## Abstract

**Background:**

Additive manufacturing (AM) technology has helped to achieve several advances in the medical field, particularly as far as fabrication of implants is concerned. But the application of direct metal laser sintering (DMLS) bone plate is quite limited due to the indeterminate mechanical property. The purposes of this study were to characterize the biomechanical properties of the polished DMLS reconstruction plate and to compare these with the properties of commonly applied implants and to find whether the mechanical performance of DMLS plate meets the requirements for clinical application.

**Methods:**

In this study, we fabricated two groups of plates by DMLS and computer numerical control (CNC) techniques. After that, we polished all samples and investigated their roughness, components, hardness, static bending, and torsional performance. Moreover, cyclic bending tests and fractographic analysis were conducted. Statistical comparisons of the group by means of monotonic test data were made, and a qualitative comparison was performed to assess failures in fatigue.

**Results:**

We found no differences in surface roughness or components after polishing, but the DMLS plate hardness is 7.42% (*p* < 0.01) greater than that of the CNC plates. Compared with the CNC plates, the DMLS plate static bending and torsional performance were significantly greater. In a dynamic test, the DMLS plates survived 10^6^, 10^6^, 32,731, and 33,264 cycles under 0.6, 0.8, 0.9, and 1 kN cyclic loads, respectively, while the CNC plates survived 10^6^, 10^6^, 10^6^, and 283,714 cycles.

**Conclusions:**

These results indicate that the mechanical performances of the DMLS plate are stronger, and the strength under fatigue tests is sufficient. DMLS implant has great potential and may become a better choice for clinical use in the future. However, direct application of these AM instruments in the operating room requires further validation including animal and clinical experiment.

## Background

Reconstruction plates play an important role in the internal fixation system because they are malleable and can be contoured to fit in nearly every direction to accommodate mismatched appositional surfaces [1–3]. These characteristics accelerate the application of reconstruction plates in clavicular fractures and scapular and pelvic fixations. However, implant failures, such as plastic deformation or breaking, have been reported. Subsequently, the strength of reconstruction plates for fixation has been questioned [4, 5]. A most common reason for fixation failure may be a result of the repeated twisting during operation in order to match the bone surfaces.

In manufacturing techniques, the traditional reconstruction plates fabricated by a reduction of material manufacturing have some disadvantages. First, the computer numerical control (CNC) technique wastes material as it discards raw material during the fabrication process. Second, the products are not suited for every patient. In contrast, additive manufacturing (AM) using direct metal laser sintering (DMLS) methods have been successfully applied to fabricate implants, which may solve the above problems. DMLS, a form of rapid prototyping (RP) technique, rapidly manufactures various complex objects layer by layer based on a computer-aided design (CAD) model. A high-power laser beam is used for DMLS procedures, which makes the production process much faster because of the metal powder that leads to a light reflex during sintering. And high-quality implants or prostheses are fully constructed using melted metal powders to ensure that the products are completely void-free. Furthermore, DMLS has been attracting great attention because it provides accurate control over internal pore architectures or complex shapes. Additionally, a full DMLS process occurs in an ultra-high-vacuum environment that may prevent defects caused by oxidation and DMLS has the advantage of fabricating components with a much lower residual stress which strongly influence fatigue life [6, 7]. Such characteristics allow for the manufacturing of advantageous tailored implants.

According to previous studies, Ti-6Al-4V is widely applied in the biomedical field because it is known to exhibit both a compression strength and elastic modulus similar to that of the human bone [8]. This significantly reduces the difference between natural bone and the implant, alleviating premature failure caused by the stress-shielding phenomenon. Harrysson et al. [9] described a new method for creating tailored hip stems with bone ingrowth surfaces from biocompatible Ti-6Al-4V using the RP technique. Jardini et al. [10] reported that DMLS-manufactured components were successfully applied to design and fabricate a biomodel and then were customized for the surgical reconstruction of a large cranial defect using Ti-6Al-4V powders. However, DMLS-printed implants for individuals require superior mechanical performance. In fact, studies on the mechanical and material properties of the DMLS products are limited. The purpose of this study was to examine the mechanical behavior of two different bone plates. We hypothesized that the DMLS and CNC plates, suffering surface post-treatment, possessed differences in various mechanical tests and sought to quantify those differences. Based on the results, we can evaluate their clinical applicability and may generate a new way to manufacture a next-generation implant to improve the fixed effect of bone fractures.

## Methods

### Fabrication of DMLS and CNC plates

We selected an eight-hole 3.5-mm reconstruction plate (length 96 mm, width 10.6 mm, thickness 3 mm) to be the object of this study. On one side, we fabricated 17 plates by the CNC technique based on a three-dimensional CAD model. The bulk material was a Ti-6Al-4V bar. On the other side, we designed a new model by expanding 1 mm in order to facilitate subsequent post-treatments and be consistent with the size of the DMLS group. Then, we input it into a three-dimensional (3D) printed system (EOSINT M290, Germany) to print the other 17 plates. The material of all plates was Ti-6Al-4V powder (Arcam AB, Sweden). Then, we measured the surface roughness by a contour roughness meter (MarSurf SD 26, Germany). Afterwards, the same surface post-processing, mainly including polishing, was applied to every plate. As a result, we fabricated 17 DMLS and 17 CNC plates undergoing the same post-treatment for comparison.

### Basic material property test

After post-processing, we measured and recorded the length, width, thickness, and hole diameter of each plate. Thereafter, surface roughness measurements, using scanning electron microscope observation via low- and high-power microscopic views, were conducted. Moreover, to determine the plate components manufactured by two different techniques, we randomly chose three plates from each group to conduct gas component analysis (LECO ONH836, USA), metal element analysis (FMP, UK), and hardness test (DHV-1000, Shanghai, China).

### Mechanical analysis

#### Monotonic four-point bending test

**Fig. 1:**
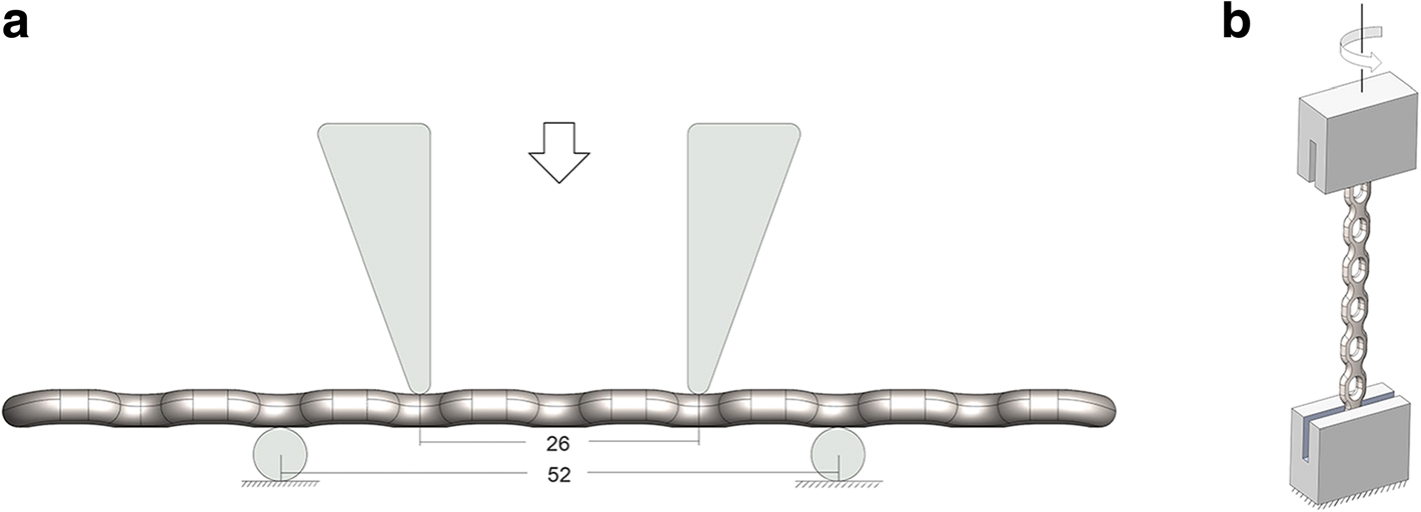
Positions of tested plates and rollers in the bending test (**a**) and torsion test (**b**)

Five plates from each group were subjected to the monotonic four-point bending test by a microcomputer control electronic universal test (MTS system, SANS, USA) to determine bending strength and stiffness. The loading and support rollers were positioned as described by ISO 9585 [11] (Fig. 1a). The plate was positioned in the four-point bending fixture with the bone interface of the plate in contact with the loading roller shaft surface. The distance between the loading rollers (k) was 13 mm and that between the support rollers (2*h* + *k*) was 26 mm. Each plate was positioned medially.

In the single cycle four-point bending test, a displacement-controlled test was used at 0.1 mm/s until 5 mm displacement was reached; the displacement and load data from sensors were used to establish the bending stiffness of each bone plate defined by the ISO 9585 standard as the maximum slope of the linear elastic portion of the load versus load-point displacement curve (N/mm). The bending moment-deflection curves were generated using Microsoft Excel (Microsoft Corporation; Redmond, WA, USA). A line that represents the slope for bending stiffness was offset by 0.56 mm (*q* = 0.02(2*h* + *k*)). The point where the offset bending stiffness slope intersected the bending moment-deflection curves was the proof load (*P*) reported in kilonewton as described by the ISO standard, and the equivalent bending stiffness (*E*) and bending strength were determined using the expression:

$$ E=\frac{\left(2\mathrm{h}+3\mathrm{k}\right){Sh}^2}{12};\kern0.5em \mathrm{bending}\  \mathrm{strength}=\frac{Ph}{2}. $$

where

*E* (N m^2^) = the equivalent bending stiffness

*k* (mm) = the center span distance

*h* (mm) = the distance between inner and outer rollers

*S* = the slope of the bending moment-deflection curve

*P* (kN) = the proof load

#### Monotonic torsion test

Five plates from each group were subjected to the monotonic torsion test. As shown in Fig. 1b, the proximal and distal end (12 mm) of each plate was clamped, driven by a material testing machine (ElectroPuls E10000, USA). We fixed the inferior clamp and rotated the upper fixture unidirectionally at a rate of 0.5°/s. The test was terminated when the implant cracked or the angle of rotation reached 160°. Torque versus degree data was recorded by the sensor, and the torque-rotation curves were generated. Three parameters of interest were measured as Eglseder et al. [12] described: (1) maximum torque, (2) torsional stiffness, and (3) maximum torque within 10° of rotation. Torsional stiffness was calculated as the slope of the torque versus the angular displacement curve between 1 N-m of torque and the subsequent 10° of angular deformation. The maximum torque within 10° was defined as the maximum torque attained between 1 N-m of torque and the subsequent 10° of deformation.

#### Cyclic bending fatigue test

In the dynamic four-point bending test, four DMLS plates and four CNC plates were successively positioned according to the monotonic four-point bending test described above to demonstrate the fatigue property. Dynamic tests were carried out with progressive compressive sinusoidal loading. Loading pattern and loading direction simulated the in vivo loading of the plate placed on the tension side.

According to the ASTM standard and considering a safety factor, the plates are specified to withstand 10^6^ load cycles. Therefore, based on the monotonic bending test and the preliminary test, four loading patterns, 0 to − 600 N, 0 to − 800 N, 0 to − 900 N, and 0 to − 1000 N, at a rate of 5 Hz, were applied to the implants in sequence. Fatigue failure was defined as a visible crack observed in the implant, and the tests were terminated after finishing one million cycles or appearing a crack.

### Statistical methods

All the data were collected and analyzed by SPSS Statistics v20 software (IBM, Armonk, NY, USA). Means and standard deviations were calculated for descriptive purposes. The presented box plots represent median and first and third quartiles. The error bars indicate maximum and minimum values. Two-tailed, unpaired Student *t* tests at a level of significance of *a* = 0.05 were used to detect significant differences; these were conducted with two samples from two independent populations. The differences in roughness were examined for significance (*p* < 0.05) using a paired samples test and a one-sample *t* test. Because fatigue testing was done on a small number of plates for each load pattern, a qualitative comparison of fatigue performance was performed.

## Results

### Basic material properties

**Fig. 2:**
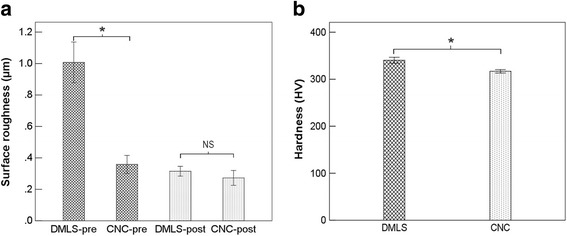
**a**, **b** Results of surface roughness and hardness of plates before and after polishing, respectively. *Indicates statistical significance (*p* < 0.05). NS indicates that the difference is not significant

The observed DMLS plate surfaces were relatively rough with an irregular texture compared with CNC plates before polishing. However, as shown in Fig. 2a, the difference in surface roughness in the two groups was not significant after performing the surface treatment. To compare with target sizes, as presented in Table 1, we found no significant difference (*p* > 0.05) between groups, implying that the DMLS and CNC manufacture techniques were able to provide adequate accuracy. And the post-treatment plate sizes were no different (*p* > 0.05). The consequences of metal and gas element analysis (Tables 2 and 3) showed that the proportions of the components of the two groups were similar and were within the scope of standard values (ISO standard). As presented in Fig. 2b and Table 4, the hardness of the DMLS plates (= 340.3333 HV) is 7.42% (*p* < 0.01) greater than another (= 316.8333 HV).

**Table 1:** Results of size measurements

	Before polishing				After polishing		
	DMLS plate		CNC plate		Target size	DMLS plate (actual size)	CNC plate (actual size)
	Target size	Actual size	Target size	Actual size			
Length	96.500	96.503	96.100	96.091	96.000	96.003	96.001
Width	11.100	11.077	10.700	10.699	10.600	10.598	10.601
Thickness	3.500	3.493	3.100	3.101	3.000	3.007	3.005
Hole diameter	4.000	4.013	3.600	3.601	3.500	3.501	3.500

All data are mean values (*n* = 15). Comparing the target size and polished plate size, we found that none of the differences between groups were significant (*p* > 0.05), implying that the DMLS manufacturing technique is able to provide adequate accuracy for bone plates used in clinical applications

**Table 2:** Results of metal element analysis

	Ti	Al	V	Cr	Cu	Fe	Mn	Mo
DMLS plate	89.5667	6.0033	3.9733	< 0.0012	0.0228	0.1787	0.0050	0.0055
CNC plate	89.6667	5.8200	4.0800	< 0.0012	0.0204	0.1440	0.0050	0.0136
	Nb	Sn	Si	Zr	Pd	Ru	C	W
DMLS plate	0.0113	0.0152	0.0123	0.0135	0.0093	0.0655	0.0122	0.0171
CNC plate	0.0126	0.0027	0.0242	0.0135	0.0206	0.0743	0.0120	0.0167

All of the components are within the scope of the standard values (%): Al 5.5~6.75, V 3.5~4.5, Fe < 0.3, and C < 0.08

**Table 3:** Gas elemental analysis

	O (%)	N (%)	H (%)
DMLS plate	0.1003	0.0219	0.0028
CNC plate	0.1156	0.0070	0.0033

All of the O, N, and H elements are within the scope of the standard values (%): O ≤ 0.20, N ≤ 0.05, and H ≤ 0.015

**Table 4:** Static four-point bending test and monodirectional torsion test outcomes

	DMLS plate	CNC plate	*p* value
*Equivalent bending stiffness (N m^2^)	1.0431 ± 0.0568	0.8633 ± 0.0844	0.004
*Bending strength (N m)	14.3743 ± 1.3103	10.3704 ± 0.8337	0.000
*Proof load (kN)	2.2113 ± 0.2015	1.5895 ± 0.1257	0.000
*Torsional stiffness (Nm/°)	0.2832 ± 0.0254	0.2442 ± 0.0115	0.014
Maximum torque within 10°(Nm)	2.8916 ± 0.2841	2.6716 ± 0.3431	0.302
*Maximum torque (Nm)	15.0258 ± 0.7897	10.9577 ± 0.4315	0.000
*Hardness (HV)	340.3333 ± 5.989	316.8333 ± 3.430	0.000

All data are presented as ^−^ Χ ± SD (*n* = 5)

*Indicates statistical significance (*p* < 0.05)

### Static tests

**Fig. 3:**
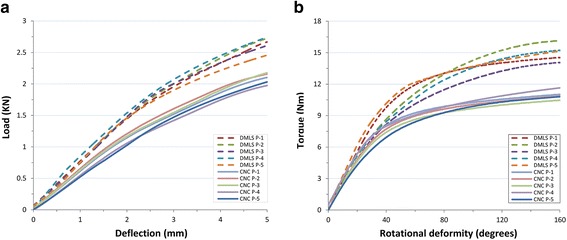
**a**, **b** Bone plate bending and torsional properties. The maximum slope (*S*) of the linear elastic portion of the load versus load-point displacement curve is generated to calculate the equivalent bending stiffness (*E*) that might be modified to better reflect the bending and torsional properties. We could intuitively confirm that the bending and torsional performances of the DMLS plates are stronger than those of the CNC plates

**Fig. 4:**
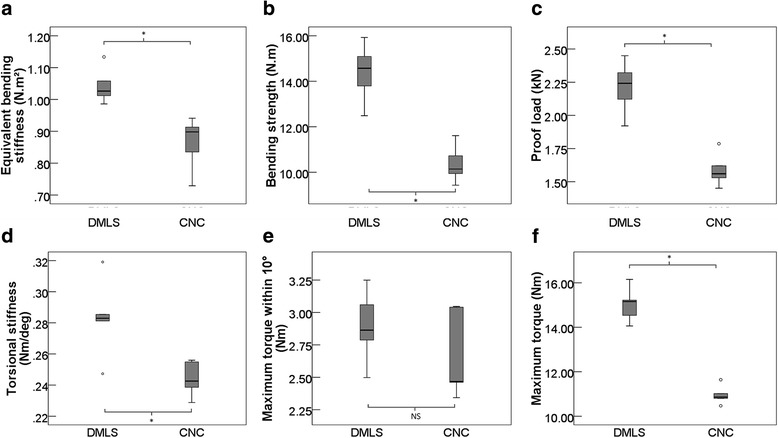
Box plots demonstrating the mechanical properties: bending strength (**a**), equivalent bending stiffness (**b**), proof load (**c**), torsional stiffness (**d**), maximum torque within 10° (**e**), and maximum torque (**f**). *Indicates statistical significance (*p* < 0.05). NS indicates that the difference is not significant

The static four-point bending test and the monodirectional torsion test outcomes are presented in Table 4. Thereafter, the load-deflection diagrams and torque-displacement curves were generated to show the trend and discrepancy (Fig. 3a, b). Comparing the load displacement curves, differences between the groups became evident. The offset displacement was 0.56 mm according to the ISO standard [11]; the DMLS plates yielded a variable displacement range of 2.2113 ± 0.2015 kN, and the CNC plates yielded a plate displacement range of 1.5895 ± 0.1257 kN. Both plates did not break under the two test patterns. We observed that the bending and torsional areas of the DMLS plates and CNC plates were between the two load rollers at the roller screw. As shown in Table 4, the equivalent bending stiffness, bending strength, and proof load of DMLS samples in the monotonic bending test were 20.83% (*p* < 0.05), 38.61% (*p* < 0.05), and 39.12% (*p* < 0.05), respectively, and were significantly stronger than those of the CNC samples (Fig. 4a–c; Table 4). Compared with the CNC plates, the torsional stiffness, maximum torque within 10°, and maximum torque of the DMLS plates during torsion test were 15.97% (*p* > 0.05), 8.23% (*p* > 0.05), and 37.13% (*p* < 0.05) greater, respectively (Fig. 4d–f; Table 4).

### Fatigue test and fractographic analysis

**Fig. 5:**
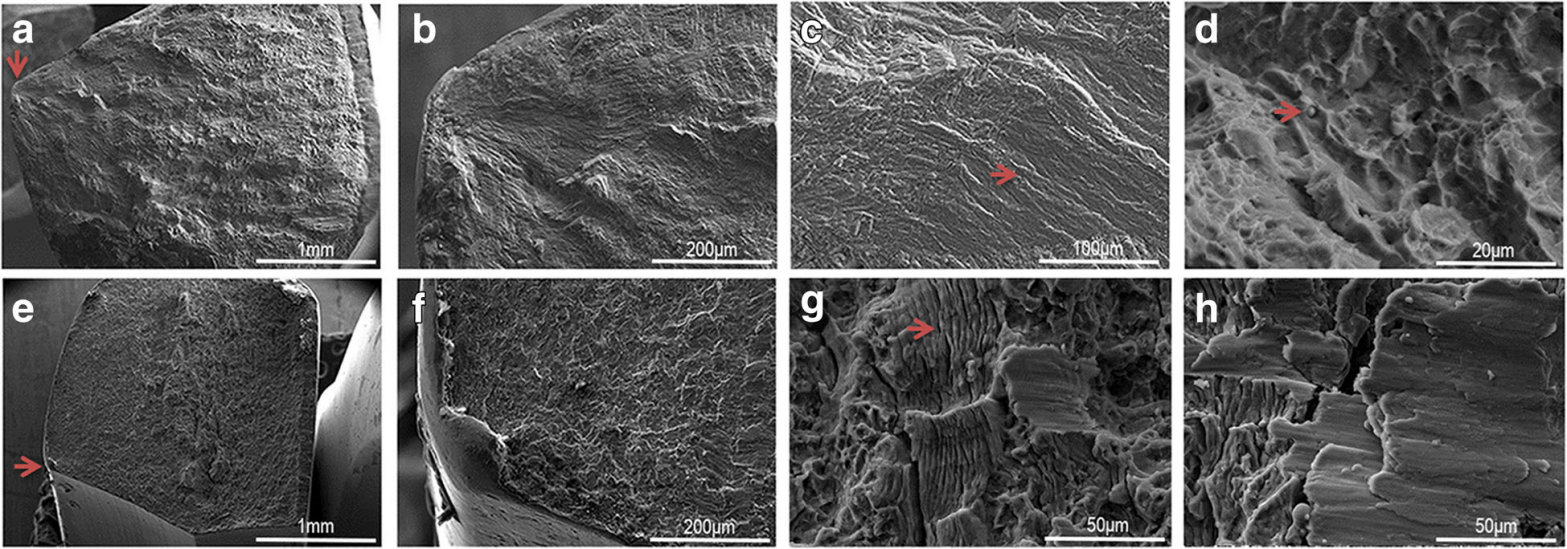
Fractographic analysis by SEM. **a**–**d** Represent the fracture surface appearance of a DMLS plate from low- to high-power lenses. **e**–**h** Represent a CNC plate

As shown in Table 5, two DMLS plates and one CNC plate failed in the fatigue test. The developed cracks in all the failed plates occurred consistently at the edge of innermost screw hole. Under 0.9 and 1 kN load pattern, CNC plates survived longer than DMLS plates. To understand the underlying fatigue mechanisms, a fractographic analysis was conducted by a scanning electron microscope (SEM). The representative fracture modes of the studied samples were presented in Fig. 5. Figure 5a, e shows an entire fracture surface at low magnification. The possible failure origin (indicated by red arrows) can be more clearly seen in Fig. 5b, f. Figure 5c, g illustrates fatigue striations that were seen near the failure origin on the fracture surface at a high magnification. Figure 5d, h represents the final fracture zone. Microvoid dimples, characteristic features of fast fractures in metals, were observed in Fig. 5d. They are far from the failure origin on the fracture surface; some unsintered powders were observed to disperse inside the dimples from the DMLS samples.

**Table 5:** Results of fatigue test

Loading pattern	DMLS plate	CNC plate
0.6 kN	10^6^ cycles	10^6^ cycles
0.8 kN	10^6^ cycles	10^6^ cycles
0.9 kN	32,731 cycles	10^6^ cycles
1.0 kN	33,264 cycles	283,714 cycles

Fatigue failure was defined as a visible crack observed in the implant; tests were terminated after finishing one million cycles or appearing a crack

## Discussion

In this study, we investigated the mechanical performance including bending, torsion, and high cyclic fatigue properties of plates that are fabricated by the DMLS and CNC techniques. The results showed that the static mechanical strength of DMLS plates was significantly greater than that of CNC plates. Although the former’s fatigue performance was slightly inferior to the latter, the fatigue strength of the DMLS plate met the requirement for clinical application.

Surface properties are a key factor that strongly impact material fatigue behavior. As a kind of AM technology, the DMLS technique manufactures implants by directly laser sintering metal layer by layer, contributing to a much rougher surface compared to that by traditional CNC technique. This is in agreement with previous reports [5, 13]. Some researchers observed the surface of implants that underwent surface post-treatments possessed a similar micro-roughness and homogeneously distributed nanostructures [6, 14]. Therefore, in order to enhance the comparability of the two groups, the same polishing method was applied to the plates, and as a result, the roughness values of the two groups showed no significance.

The plate ingredients were derived from titanium alloy powders and bars. Because of the differences in the two manufacturing methods, it is necessary to contrast their compositions. Based on the results, it seems that the influence of two different manufacturing methods on the gas and metal composition are consistent. Additionally, the proportions of components were both within the scope of standard values and were adequate for clinical use requirements.

Mechanical tests were the main criteria in this study. Our study design followed the ISO and ASTM standard test method [11] for static bending and torsion properties of bone plates. As shown in Fig. 4 and Table 4, the monotonous bending and torsion performances of DMLS plates were significantly stronger than those of CNC plates used clinically. The DMLS curve exhibit a steeper slope both in bending and torsion tests (Fig. 3a, b). The same curves were reported by Liu PC et al. [5] and DeTora et al. [15]. Their bending test results are similar to ours; however, the data of torsion test are not reported. Aluede et al. [16] and Overturf et al. [17] examined the bending and torsional stiffness of an eight-hole plate with data differing from ours. We think that the difference may result from the different loading conditions, plate types, and sizes. Additionally, the mean hardness of 3D-printed plates reported by Liu PC et al. [5] was 341.1 HV10 ± 1.93, which is strongly consistent with ours.

The fatigue property of implants is quite important during the healing period when there is a lack of bony support [16]. In this study, the conditions for fatigue testing were designed to reflect the conditions during fracture healing that normally occurs in 3 to 4 months postoperatively. The 10^6^ cycle testing limit is based on an estimate of 5000 to 14,000 load cycles per day during the approximate 12 to 16 weeks required for fracture healing [16]. It was estimated that 0.5 to 1.6 million loading cycles might occur during the time required to achieve bone union [18]. In most cases where permanent deformation occurred and in all cases where fatigue fracture was observed, failure was encountered at less than one million cycles. Furthermore, the loading level used in this study was higher than in previous studies [19–21]. Therefore, the fatigue performance of the DMLS plate is sufficient for clinical application.

In the bending fatigue test, both tensile and compressive stresses are present in the structure and the fatigue crack growth becomes an important factor influencing the fatigue failure. On the fracture surface, it could be observed that the initiations of the fatigue crack are both at the screw hole adjacent to the loading roller. Manufacturing technique and surface post-treatments can significantly influence implant fatigue life, as has been previously reported [22].

The fatigue crack has been attributed to nonmetallic inclusion bodies embedded in the surface, perhaps because of processing [18]. In this case, the fatigue crack is more prone to initiate and propagate from a rough surface or internal defects because of higher local tensile stress by structure bending. Near the failure origin, the fatigue striation tendency was in disorder. That might attribute to the increased number of pores and not fully sintered powders compared to CNC plates. Figure 5c, g illustrates fatigue striations seen near the failure origin on the fracture surface. They are created as the crack tip advances because of subcritical crack growth during cyclic loading. In addition, microvoid dimples, a characteristic feature of a fast fracture, were evident far from the failure origin on the fracture surface.

Previous studies have investigated fatigue properties of metal alloys fabricated using AM techniques [14, 23] and put forward some other methods to improve the fatigue life of implants [24–26]. Thus, the 3D-printed plate has significant potential to improve and perform well, although its fatigue behavior still remains flaws.

Our study investigated the primary mechanical and material properties of DMLS and conventional CNC plates. The advantages of the AM technique are obvious. Personalized customization is an important aspect. It can offer a perfect matching in an irregular bony surface and plate, which avoids repeated pre-bending and screw hole deformation. This is quite important to ensure the effect of fixation. In addition, individual anatomic 3D-printed plate can reduce the adverse impact of screws that leads to difficult fixation. Moreover, 3D-printed technique could be used to fabricate complicated porous structure. That may be difficult to finish in the traditional process. The results of our research could offer a mechanical reference to extend its clinical application.

However, this study has some limitations. First, the conclusions are limited because of the small number of samples. Second, the torsional fatigue behavior test of DMLS implants is absent because of the inferior fixture. Moreover, a fracture fixation scenario and the biosecurity and biocompatibility tests should be considered.

## Conclusions

These results indicate that the mechanical performances of the DMLS plate are stronger than those of the CNC plate. And the strength under fatigue tests is sufficient. DMLS implant, as an AM technique, has great potential and may become a better choice for clinical use in the future. However, direct application of these AM instruments in the operating room requires further validation including animal and clinical experiment.

## Abbreviations

AM: Additive manufacturing
CAD: Computer-aided design
CNC: Computer numerical control
DMLS: Direct metal laser sintering
FGM: Functionally graded structure
RP: Rapid prototyping

## References

[1] Azuma M, Yanagawa T, Ishibashi-Kanno N, Uchida F, Ito T, Yamagata K (2014). Mandibular reconstruction using plates prebent to fit rapid prototyping 3-dimensional printing models ameliorates contour deformity. Head Face Med.

[2] Chung KJ, Hong DY, Kim YT, Yang I, Park YW, Kim HN (2014). Preshaping plates for minimally invasive fixation of calcaneal fractures using a real-size 3D-printed model as a preoperative and intraoperative tool. Foot Ankle Int.

[3] Ruedi TP, Buckley R (2007). AO principles of fracture management Vol 2 specific fractures.

[4] Woltz S, Duijff JW, Hoogendoorn JM, Rhemrev SJ, Breederveld RS, Schipper IB (2016). Reconstruction plates for midshaft clavicular fractures: a retrospective cohort study. Orthop Traumatol Surg Res.

[5] Liu PC, Yang YJ, Liu R, Shu HX, Gong JP, Yang Y (2014). A study on the mechanical characteristics of the EBM-printed Ti-6Al-4V LCP plates in vitro. J Orthop Surg Res.

[6] Hyzy SL, Cheng A, Cohen DJ, Yatzkaier G, Whitehead AJ, Clohessy RM (2016). Novel hydrophilic nanostructured microtexture on direct metal laser sintered Ti-6Al-4V surfaces enhances osteoblast response in vitro and osseointegration in a rabbit model. J Biomed Mater Res A.

[7] Lin WS, Starr TL, Harris BT, Zandinejad A, Morton D (2013). Additive manufacturing technology (direct metal laser sintering) as a novel approach to fabricate functionally graded titanium implants: preliminary investigation of fabrication parameters. Int J Oral Maxillofac Implants.

[8] Parthasarathy J, Starly B, Raman S, Christensen A (2010). Mechanical evaluation of porous titanium (Ti6Al4V) structures with electron beam melting (EBM). J Mech Behav Biomed.

[9] Harrysson OLA, Cansizoglu O, Marcellin-Little DJ, Cormier DR, Ii HAW (2008). Direct metal fabrication of titanium implants with tailored materials and mechanical properties using electron beam melting technology. Mater Sci Eng C.

[10] Jardini AL, Larosa MA, Maciel FR, Zavaglia CA, Bernardes LF, Lambert CS (2014). Cranial reconstruction: 3D biomodel and custom-built implant created using additive manufacturing. J Craniomaxillofac Surg.

[11] Cesarone DM, Disegi JA. Techniques in the application of ISO 9585 test method for the determination of bone plate bending properties. Astm Spec Tech Publ. 1993;7

[12] Eglseder WA, Jasper LE, Davis CW, Belkoff SM (2003). A biomechanical evaluation of lateral plating of distal radial shaft fractures. J Hand Surg Am.

[13] Hrabe NW, Heinl P, Flinn B, Korner C, Bordia RK (2011). Compression-compression fatigue of selective electron beam melted cellular titanium (Ti-6Al-4V). J Biomed Mater Res B Appl Biomater.

[14] Guan B, Wang H, Xu R, Zheng G, Yang J, Liu Z (2016). Establishing antibacterial multilayer films on the surface of direct metal laser sintered titanium primed with phase-transited lysozyme. Scientific Reports..

[15] Detora M, Kraus K (2008). Mechanical testing of 3.5 mm locking and non-locking bone plates. Vet Comp Orthop Traumatol.

[16] Aluede E, McDonald E, Jergesen H, Penoyar T, Calvert K (2014). Mechanical behaviour of low-cost dynamic compression plates correlates with manufacturing quality standards. Int Orthop.

[17] Overturf SJ, Morris RP, Gugala Z (2014). Biomechanical comparison of bicortical locking versus unicortical far-cortex-abutting locking screw-plate fixation for comminuted radial shaft fractures. J Hand Surg.

[18] Kanchanomai C, Phiphobmongkol V, Muanjan P (2008). Fatigue failure of an orthopedic implant—a locking compression plate. Eng Fail Anal.

[19] Eden L, Doht S, Frey SP, Ziegler D, Stoyhe J, Fehske K (2012). Biomechanical comparison of the locking compression superior anterior clavicle plate with seven and ten hole reconstruction plates in midshaft clavicle fracture stabilisation. Int Orthop.

[20] Robertson C, Celestre P, Mahar A, Schwartz A (2009). Reconstruction plates for stabilization of mid-shaft clavicle fractures: differences between nonlocked and locked plates in two different positions. J Shoulder Elbow Surg.

[21] Drosdowech DS, Manwell SE, Ferreira LM, Goel DP, Faber KJ, Johnson JA (2011). Biomechanical analysis of fixation of middle third fractures of the clavicle. J Orthop Trauma..

[22] Azevedo CRF, Hippert E (2002). Failure analysis of surgical implants in Brazil. Eng Fail Anal.

[23] Bandyopadhyay A, Espana F, Balla VK, Bose S, Ohgami Y, Davies NM (2010). Influence of porosity on mechanical properties and in vivo response of Ti6Al4V implants. Acta Biomater.

[24] Zhao S, Li SJ, Hou WT, Hao YL, Yang R, Misra RD (2016). The influence of cell morphology on the compressive fatigue behavior of Ti-6Al-4V meshes fabricated by electron beam melting. J Mech Behav Biomed Mater.

[25] Li S, Zhao S, Hou W, Teng C, Hao Y, Li Y (2016). Functionally graded Ti-6Al-4V meshes with high strength and energy absorption. Adv Eng Mater.

[26] Li SJ, Murr LE, Cheng XY, Zhang ZB, Hao YL, Yang R (2012). Compression fatigue behavior of Ti-6Al-4V mesh arrays fabricated by electron beam melting. Acta Mater.

